# Removal of Cefixime from Water Using Rice Starch by Response Surface Methodology

**Published:** 2020

**Authors:** Fatemeh Sadat Tabatabaei, Mahdi Asadi-Ghalhari, Rahim Aali, Fatemeh Mohammadi, Roqiyeh Mostafaloo, Rezvaneh Esmaeili, Zohreh Davarparast, Zahra Safari

**Affiliations:** Department of Environmental Health Engineering, Faculty of Health, Qom University of Medical Sciences, Qom, Iran

**Keywords:** Cefixime, Response surface methodology (RSM), Rice starch

## Abstract

**Background:**

Remaining pharmaceutical compounds cause environmental pollution. Therefore, refining these compounds has become a major challenge. In this study, the function of eliminating Cefixime (CFX) using rice starch was evaluated under controlled conditions.

**Methods:**

Response Surface Methodology (RSM) was used to design, analyze, and optimize experiments, and the interaction between four variables including pH (3–9), rice starch dose (0–300 *mg/L*), CFX initial concentration (0–16 *mg/L*) and time (20–120 *min*) was investigated on CFX removal.

**Results:**

The optimum pH, starch dose, initial concentration and time were 4.5, 225 *mg/L*, 7.9 *mg/L* and 95 *min*, respectively. The maximum efficiency of CFX removal was 70.22%. According to RSM, this study follows a quadratic model (R^2^=0.954).

**Conclusion:**

Rice starch has been successful in removing CFX from the aqueous solution. Therefore, it is recommended to utilize this process to remove CFX from aqueous solutions.

## Introduction

In recent years, pharmaceutical compounds have been considered as the most serious water pollutants ^1^. One of the most important groups of pharmaceuticals are antibiotics that are used to prevent and treat infections of farms, cattles and human ^2^. Water pollution is the result of uncontrolled use and a serious environmental threat, which may lead to creation of antibiotic-resistant microbes and pose a serious threat to all humanity in coming years ^3^. Also, these compounds are toxic for aquatic organisms even at low concentrations ^4^. The excretion of active and unchanged compounds has been proven in many antibiotics ^5^. Since removing these compounds entirely is not feasible in conventional wastewater treatment plants, these compounds enter into surface water, groundwater, and water treatment plants ultimately, and as a result of not being removed from treatment plant, they enter drinking water ^6^.

**Figure 1.**
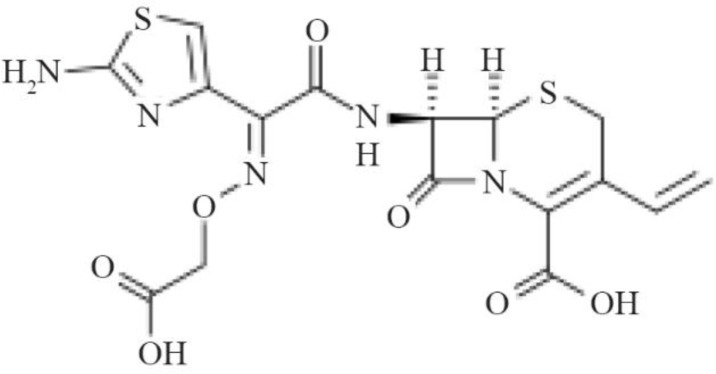
Cefixime molecular structure.

One of the most important antibiotics utilized to treat infection is cefixime. Cefixime is a semi-synthetic antibiotic from the third generation of cephalosporins. Cefixime can be consumed against a variety of bacterial organisms and infections including Staphylococci, Influenza, Hemophilia, *Escherichia coli*, Streptococcus, fever and chills, and throat infections ^7,8^. Studies show this compound has been one of the most widely consumed drugs in Europe in recent years ^9^. The molecular structure of Cefixime is demonstrated in figure 1 ^10^.

Due to persistent incapability of conventional waste-water treatment methods to eliminate or diminish the number of undesired antibiotics, several more efficient treatment methods ^11^ based on advanced oxidation, ion exchange and adsorption with activated carbon, reverse osmosis and biological treatment are applied among which biological treatment is not very effective in removing antibiotics ^7^. The adsorption process is extensively used to remove organic contaminants. This method has an advantage over other chemical methods due to the lack of metabolite production. The adsorption process has received much attention compared to other purification techniques regarding initial cost, reuse of effluent, simplicity, and flexibility in design, easy operation and non-sensitivity to pollutants and toxic compounds. Studies have proven that adsorption process is advantageous for removing antibiotics from aqueous environments ^12^. In many studies, rice starch has been described as a highly efficient adsorbent to remove heavy metals such as zinc, lead, copper, and cadmium ^13^. The average size of rice starch granules is 3–8 *μl* and they are angular and polyhedral. Amylopectin is the dominant component in rice starch and plays a significant role in its physico-chemical properties. Rice starch has more priorities than other starches due to its characteristics such as high stability, high acid resistance and wide range of amylase to amylopectin ratio ^14^. Owing to water hygiene protection and human protection against severe health consequences of antibiotics, these compounds must be effectively and appropriately removed from sewage and water resources. The use of natural materials is of great importance in eliminating pollutants such as antibiotic compounds from an environmental point of view.

Studies have shown that the process of removing cefixime from water sources with rice starch has not been investigated so far. In this study, removal of cefixime with rice starch was performed through Response Surface Methodology (RSM). RSM is a statistical method for designing experiments that is used to optimize, evaluate the interaction of independent factors and reduce the number of experiments in chemical and biochemical processes ^15^. The objective of using RSM is to identify optimal operating conditions or to enhance operating conditions ^16^. In this study, four independent variables including CFX initial concentration, pH, starch dose and time were selected and evaluated at five levels (−α, −1, 0, +1 and +α). Design Expert software version 12 was used to design the experiment.

## Materials and Methods

### Chemicals and equipment

All the chemicals used in this study had a laboratory grade. Cefixime (CFX) was prepared with chemical formula of C_16_H_15_N_5_O_7_S_2_ and a purity of 0.98% from Sigma-Aldrich. Rice starch, HCL and NaOH were prepared from Merck. 1 *g/L* CFX stock was made and intended concentrations were obtained from it. In this study, 3% starch solution was prepared using deionized water. To prevent hydration of starch solution during experiments, the solution was freshly prepared. Stoke solution with suitable dilution ratio was used to make other concentrations used in this study. Evaluating CFX removal function by rice starch was the experiment design which was modeled and optimized using Design Expert software (Version 12). The Central Composite Design (CCD) based on RSM was used to find the optimal conditions, the effect of parameters and their interaction in CFX removal by starch. In this study, CCD with four variables including pH, starch dose, CFX initial concentration and time, at five levels (−α, −1, 0, +1, +α) was chosen. According to the equation 2k+2k+C, total number of experiments designed was 30 runs which K as the number of factors and C as the number of central points ^17^. Tables 1 and 2 show process independent variables, their values and experiment design matrix. The one-way analysis of variance (ANOVA) was utilized to determine adequacy of the proposed model, to determine R^2^, adjusted R^2^ and predicted R^2^ ^18^.

**Table 1.** Levels of independent variables in experiment design

**Factor (Unit)**	**Code**	**Level**				

		**−α**	**−1**	**0**	**+1**	**+α**
**pH**	X_1_(A)	3	4.5	6	7.5	9
**Starch dose (*mg/L*)**	X_2_(B)	0	75	150	225	300
**Initial CFX concentration (*mg/L*)**	X_3_(C)	0	4	8	12	16
**Retention time (*min*)**	X_4_(D)	20	45	70	95	120

**Table 2.** Experimental design matrix used to evaluate the rate of CFX removal by rice starch

**Std order**	**pH**	**Starch dose (*mg/L*)**	**Initial CFX concentration (*mg/L*)**	**Retention time (*min*)**	**% Removal**		

					**Obtained**	**Predicted**	**Residual**
**1**	4.5	75	4	45	43.00	35.42	83.92
**2**	7.5	75	4	45	39.48	38.06	2.16
**3**	4.5	225	4	45	57.49	65.16	42.91
**4**	7.5	225	4	45	47.40	51.70	−5.67
**5**	4.5	75	12	45	51.66	60.35	−17.46
**6**	7.5	75	12	45	70.00	89.89	51.26
**7**	4.5	225	12	45	64.28	80.62	23.27
**8**	7.5	225	12	45	73.22	94.07	80.31
**9**	4.5	75	4	95	35.08	39.26	−74.97
**10**	7.5	75	4	95	35.96	27.20	58.31
**11**	4.5	225	4	95	66.33	84.18	30.32
**12**	7.5	225	4	95	51.49	56.02	22.80
**13**	4.5	75	12	95	50.48	47.78	87.24
**14**	7.5	75	12	95	51.80	62.63	−37.58
**15**	4.5	225	12	95	64.57	83.24	3.17
**16**	7.5	225	12	95	63.69	81.98	−2.34
**17**	3	150	8	70	52.67	65.02	−45.74
**18**	9	150	8	70	53.68	66.40	−41.16
**19**	6	0	8	70	45.09	50.54	−32.98
**20**	6	300	8	70	69.64	99.63	−53.93
**21**	6	150	0	70	0.00	37.03	−36.43
**22**	6	150	16	70	46.47	54.59	−50.48
**23**	6	150	8	20	60.28	83.58	−86.89
**24**	6	150	8	120	60.50	75.33	−0.02
**25**	6	150	8	70	62.92	76.38	38.04
**26**	6	150	8	70	61.16	76.38	2.63
**27**	6	150	8	70	62.26	76.38	24.69
**28**	6	150	8	70	62.04	76.38	20.26
**29**	6	150	8	70	59.84	76.38	−23.52
**30**	6	150	8	70	57.86	76.38	−62.11

### Adsorption experiments

To determine relationship between four independent factors of pH (A), starch dose (B), initial CFX concentration (C) and retention time (D) in CFX removal by rice starch, CCD was used. The experiments were performed in 30 *ml* Erlenmeyer flasks at initial CFX concentrations, and pH, starch dose and retention time were designed by Design Expert. To separate starch from solution, the samples were centrifuged at 4000 *rpm* for 10 *min*. The pH of the samples was adjusted using 0.1 N solutions of either HCl or NaOH and measured by pH meter (Inolab pH=7110 model, WT-W). CFX concentration was determined using spectrophotometer (Cecil 7250 Model) at a wavelength of 288.5 *nm*. Finally, the removal efficiency of CFX was calculated by Eq.1:

(1) $$\text{RE}\;(\%)\;=\;\left[\left({\text{C}}_{0}-{\text{C}}_{\text{t}}\right)/{\text{C}}_{0}\right]/100\%$$

Where RE is the removal efficiency of CFX (%), and C0 and Ct show initial and final concentrations of CFX (*mg/L*), respectively.

## Results

### Statistical analysis

Based on statistical analysis (Table 3), one-way analysis of variance (ANOVA) shows the significance of model in elimination of CFX by different parameters (pH, starch dose, initial CFX concentration and retention time). Also, based on F-value (22.41), the model was found to be significant which means only 0.01% of data variables are likely not to be considered by the model. The Prob>F value was less than 0.05, which means the model statements are significant. The F-value for Lack of Fit in this study was 4.18, demonstrating that the relationship between Lack of Fit and pure error was not significant. In this study, the terms B, C, CD, BD, AD, AC, AB, C^2^ were significant (p< 0.05). The results obtained for the analysis of cefixime removal are presented in table 3. The values of regression coefficient (R^2^) and adjusted regression coefficient (Adj. R^2^) for the predicted quadratic model were 0.954 and 0.911, respectively.

**Table 3.** One-way analysis of variance (ANOVA) for CFX removal by rice starch

**Source**	**Sum of squares**	**df**	**Mean square**	**F-value**	**p-value Prob >F**	
**Model**	1347602	14	96257.26	22.41	< 0.0001	Significant
**A-pH**	286.1759	1	286.1759	0.07	0.7998	
**B-starch**	361573.9	1	361573.9	84.17	< 0.0001	
**C-concentration**	388483.1	1	388483.1	90.44	< 0.0001	
**D-time**	10214.37	1	10214.37	2.38	0.1439	
**AB**	25923.76	1	25923.76	6.03	0.0267	
**AC**	72401.92	1	72401.92	16.85	0.0009	
**AD**	21600.35	1	21600.35	5.03	0.0405	
**BC**	8960.239	1	8960.239	2.09	0.1692	
**BD**	23054.48	1	23054.48	5.37	0.0351	
**CD**	26902.82	1	26902.82	6.26	0.0244	
**A^2**	19519.67	1	19519.67	4.54	0.0500	
**B^2**	286.1087	1	286.1087	0.07	0.7999	
**C^2**	382438.1	1	382438.1	89.03	< 0.0001	
**D^2**	1625.161	1	1625.161	0.38	0.5477	
**Residual**	64435.39	15	4295.693			
**Lack of fit**	57550.4	10	5755.04	4.18	0.0640	Not significant
**Pure error**	6884.991	5	1376.998			
**R-squared**				0.9544		
**Adj R-squared**				0.9118		
**Pred R-squared**				0.7582		
**Adeq precision**				20.700		

The regression coefficient predicted by the model (Pred. R^2^) was also equal to 0.758 which indicates experimental values were consistent with predicted value of the model. The value of Adeq precision in predicted quadratic model was 20.7 which is a value greater than 4, indicating the accuracy of the predicted model. According to table 3, the maximum to minimum response ratio is greater than 3. Hence, to improve the model and consider the most appropriate power transmission function on the response, the Box-Cox test was applied. The Box-Cox diagram brings the data distribution closer to normal distribution. In this test, the best values of Lambda and constant K were chosen to be 1.61 and 0.73, respectively. Therefore, the improved model is demonstrated as a quadratic equation in Eq. 2:

(2) $$\begin{array}{l} {\left(\text{R}+0.73\right)}^{1.61}=+763.81+3.45\;*\text{A}+122.74\;*\text{B}+127.23\;*\text{C}-\\ 20.63\;*D-40.52\;*A\;*B+67.27\;*A\;*C-36.74\;*A\;*D-\\ 23.66\;*\text{B}\;*\text{C}+37.96\;*\text{B}\;*\text{D}-41.01\;*\text{C}\;*\text{D}-26.68\;*{\text{A}}^{2}-\\ 3.23\;*{\text{B}}^{2}-118.08\;*{\text{C}}^{2}+7.70\;*{\text{D}}^{2} \end{array}$$

### Effect of process parameters on CFX removal efficiency

**Figure 2.**
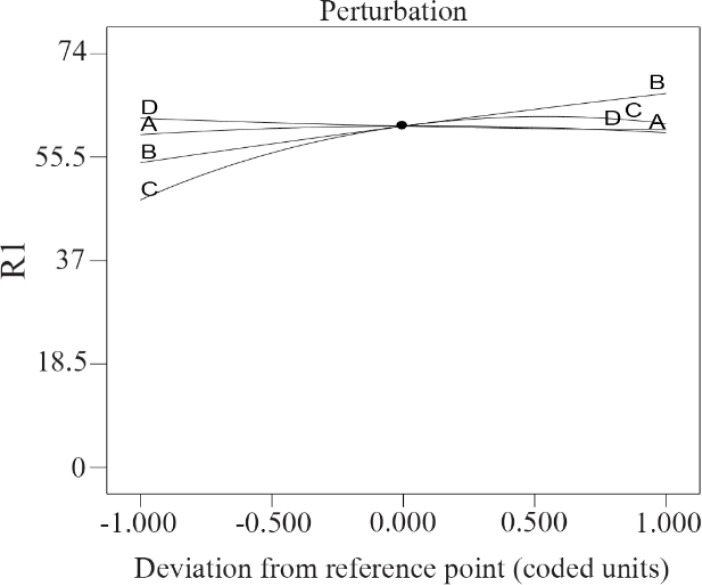
Perturbation plots for the effect of CFX removal. A) PH, B) starch dose, C) initial CFX concentration, D) retention time.

3D contours and perturbation plot were evaluated to assess the effect of variables on CFX removal efficiency using rice starch. As figure 2 shows, the perturbation plot was used to compare the effect of independent factors at a specific point in the design location. In this graph, the slope of each line indicates the effect of that factor on the amount of pollutant removal. In this plot, factors B (Starch dose) and C (Initial CFX concentration) were most effective. According to Eq 2, the initial CFX concentration with a coefficient of 127.23 had the most positive effect on CFX removal.

**Figure 3.**
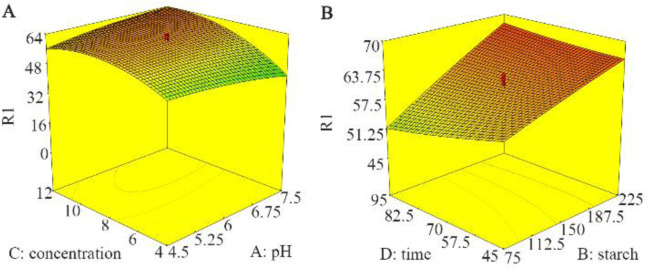
Response surface plots, removing CFX, A) pH and initial CFX concentration, B) starch dose and retention time.

3D contours in figures 3A and B show the interactions between pH, initial CFX concentration, starch dose and retention time in CFX removal rate. Figure 3A shows the interaction between the initial CFX concentration and pH. As the initial CFX concentration increased to 10 *mg/L*, the rate of CFX removal increased and then it slowed down. By reducing the pH from 7.5 to 4.5, the amount of pollutant removal increased. As figure 3B shows, the removal rate increased by increasing starch dose and reducing contact time from 95 *min* to 45 *min*.

## Discussion

According to figure 3A, the efficiency of CFX removal increased with decreasing pH. Since surface charge of CFX ions depends on pH value and pKa value of cefixime is 3.73 and 2.1 (The presence of two carboxyl groups), at pH equal to 4.5, CFX is mainly in the form of negative ions ^19^. In the present study, the results showed that with acidification of experimental environment, CFX removal was better performed. Mostafaloo *et al* ^6^ obtained similar results in the study of CFX removal by BiFeO_3_ photo catalyst.

Reducing efficiency of removal at higher CFX concentrations (Figure 3A) can be attributed to saturation and reduction of active sites on the starch surface ^6^. In a study by Dehghani *et al* ^20^ at concentrations of 0.47–0.79 *mμ* of sulfadiazine, increasing antibiotic initial concentration reduced efficacy. The same results were obtained in the study of Yoousefian *et al* ^21^, and Ouaissa *et al* ^22^.

According to the results, with increasing starch dose, the amount of pollutant removal has been increased. Therefore, it can be said increasing adsorption mass is associated with an increase in surface and more active sites for pollutant adsorption ^23^. Fakhri ^24^ in a study on efficacy of CFX removal with MGO nanoparticles, found that increasing the adsorbent dose to 0.45 *g/L* increases the efficacy of CFX removal and then the upward trend greatly slows down. Therefore, 0.45 *g/L* was selected as the optimal dose of MGO. Similar conclusions were made in the study of Elmolla ^25^ and Mostafaloo *et al* ^6^.

In this study, the CFX removal rate decreased with increasing time. Rapid adsorption at first contact time is due to large number of active sites on the adsorbent surface, which improves diffusion of cefixime to adsorbent surface ^26,27^. This result is in good agreement with Homem *et al* ^28^ and Mostafaloo *et al's* ^29^ investigations.

## Conclusion

In this study, CFX removal was performed using rice starch. The response surface methodology and CCD were used to design, analyze and optimize experiments. ANOVA showed the proposed regression model was consistent with statistical analysis: R^2^=0.9544, R^2^ adjusted=0.9118, R^2^ predicted=0.7582.

The optimal values in this study were obtained as follows: maximum CFX removal efficiency, 70.22% with desirability=0.936, optimal pH=4.5, starch dose=225 *mg/L*, initial CFX concentration=7.9 *mg/L* and time=95 *min*.

Due to proper removal of CFX with starch, it is recommended to use the sewage of industries that contain starch in pre-treatment of pharmaceutical industry wastewater.
